# Assessment of non‐vitamin K antagonist oral anticoagulants for the management of left ventricular thrombus

**DOI:** 10.1002/clc.23553

**Published:** 2021-04-02

**Authors:** Shunhui Li, Yuqing Deng, Yifan Tong, Qiangzhen Xiong, Jing Hu, Xiaojie Jiang, Tao He, Liyun Liu, Hui Chen

**Affiliations:** ^1^ Department of Cardiovascular Medicine The Third Affiliated Hospital of Nanchang University Nanchang China; ^2^ Nursing department The Third Affiliated Hospital of Nanchang University Nanchang China

**Keywords:** anticoagulants, effectiveness, safety, ventricular thrombus, warfarin

## Abstract

Although several studies have assessed the effect of non‐vitamin K antagonist oral anticoagulants (NOACs) relative to that of vitamin K antagonists (VKAs) in patients with left ventricular thrombus, the results remain controversial. Herein, a meta‐analysis was performed to compare the effectiveness and safety of NOACs versus VKAs for the treatment of left ventricular thrombus. We systematically searched the Cochrane Library, PubMed and Embase databases until November 2020 for studies that compared the effects of NOACs versus VKAs in patients with left ventricular thrombus. The treatment effects were expressed as odds ratios (ORs) with 95% confidence intervals (CIs) and pooled by a random‐effects model. Seven retrospective studies involving 865 patients with left ventricular thrombus (266 NOAC and 599 VKA users) were included. The pooled analysis suggested no difference in the rate of thrombus resolution between the NOAC and VKA groups (OR = 0.83, 95% CI 0.61–1.13). There were also no differences in the rates of stroke or systemic embolism (OR = 0.62, 95% CI 0.20–1.97), bleeding events (OR = 0.73, 95% CI 0.37–1.45), or all‐cause death (OR = 0.92, 95% CI 0.50–1.69) between patients treated with NOACs and those treated with VKAs. In addition, the rates of thrombus resolution, stroke or systemic embolism, bleeding events, and all‐cause death between NOAC‐ and warfarin‐treated patients were also similar. Our current evidence suggested that NOAC and VKA users had similar rates of thrombus resolution and clinical outcomes among patients with left ventricular thrombus. Further large‐scale prospective studies should confirm our results.

## 1 INTRODUCTION

Left ventricular thrombus is a complication of cardiac diseases such as acute myocardial infarction, heart failure, and nonischemic cardiomyopathy.^1^, ^2^, ^3^ With the rapid development of percutaneous coronary intervention, the incidence of left ventricular thrombus caused by acute myocardial infarction has decreased in recent years. In contrast, heart failure with reduced ejection fraction is currently the main cause of left ventricular thrombus. Several studies have demonstrated that left ventricular thrombus is associated with increased risk of stroke or systemic embolism and substantial morbidity and mortality.^4^ Therefore, patients with left ventricular thrombus often require anticoagulation therapy. Vitamin K antagonists (VKAs), such as warfarin, are recommended by expert consensus and guidelines and are clinically used for anticoagulation therapy in patients with left ventricular thrombus.^5^ However, VKAs have several shortcomings, including marked inter‐ and intra‐individual variations in medication dosage, a narrow therapeutic window, frequent international normalized ratio monitoring, and many drug–drug or drug–food interactions.^6^ In recent years, non‐vitamin K antagonist oral anticoagulants (NOACs) have been introduced for stroke prevention in patients with atrial fibrillation (AF). Novel drugs, including one direct thrombin inhibitor (dabigatran) and three direct Xa inhibitors (rivaroxaban, apixaban, and edoxaban), could improve the disadvantages of VKAs mentioned above. Evidence from randomized clinical trials and observational studies consistently demonstrated that NOACs are at least as effective as VKAs for stroke prevention and sometimes have better improved safety profiles in Asian or non‐Asian patients with AF.^7^, ^8^, ^9^, ^10^, ^11^ As such, NOACs have currently been recommended as first‐line oral anticoagulants in the AF guidelines.^12^, ^13^, ^14^, ^15^

However, the effectiveness and safety of off‐label use of NOACs to treat left ventricular thrombus are still unclear.^16^ Several previous reviews qualitatively described that off‐label use of NOACs could be a reasonable and valid option for the treatment of left ventricular thrombus.^5^, ^17^ However, these studies did not compare the effectiveness and safety of NOACs and VKAs in treating left ventricular thrombus. Moreover, there are still no direct head‐to‐head randomized clinical trials for this purpose. In recent years, several observational studies have assessed the effect of NOACs relative to that of VKAs in patients with left ventricular thrombus, but the results remain contradictory.^18^, ^19^, ^20^, ^21^, ^22^, ^23^, ^24^ Therefore, we quantitatively performed a meta‐analysis of observational studies comparing the effectiveness and safety of NOACs and VKAs on the rates of thrombus resolution and clinical outcomes in patients with left ventricular thrombus.

## 2 METHODS

As described previously, our current meta‐analysis was conducted according to Cochrane methodological standards, and the presentations were performed under the preferred reporting items for reporting systematic reviews and meta‐analyses.^6^ Ethical approval was not necessary because no patients were involved in setting the research question, the outcome measures, the design, or the implementation of this meta‐analysis. The data, methods, and materials of this meta‐analysis are available to others for purposes of reproducing the results or replicating procedures by contacting the corresponding author.

### 2.1 Literature search strategy

Two reviewers systematically searched the Cochrane Library, PubMed and Embase databases until November 2020 for studies that compared the effectiveness and/or safety of any NOAC (dabigatran, rivaroxaban, apixaban, or edoxaban) with that of VKAs in patients with ventricular thrombus. The following key words and their similar search terms were combined using the Boolean operator “and”: (1) ‘ventricular thrombus’ OR ‘intraventricular thrombus’ OR ‘ventricular thrombi’; (2) ‘non‐vitamin K antagonists’ OR ‘NOAC’ OR ‘new oral anticoagulants’ OR ‘novel oral anticoagulants’ OR ‘direct oral anticoagulants’ OR ‘DOAC’ OR ‘oral thrombin inhibitors’ OR ‘oral factor Xa inhibitors’ OR ‘dabigatran’ OR ‘rivaroxaban’ OR ‘apixaban’ OR ‘edoxaban’; and (3) ‘vitamin K antagonists’ OR ‘VKA’ OR ‘coumadin’ OR ‘acenocoumarol’ OR ‘phenprocoumon’ OR ‘warfarin’. In addition, we further searched the reference lists of the included studies to identify additional studies. We did not apply any restriction on the language of publication.

### 2.2 Inclusion and exclusion criteria

Studies were eligible if they met the following criteria: (1) design of the study: observational prospective or retrospective study; (2) study population: patients with ventricular thrombus, regardless of the etiology, such as heart failure with reduced ejection fraction, acute myocardial infarction, and nonischemic cardiomyopathy; (3) comparisons: any NOAC (dabigatran, rivaroxaban, edoxaban, or apixaban; any dose) versus VKAs (e.g., coumadin, acenocoumarol, phenprocoumon, and warfarin); (4) clinical outcomes: thrombus resolution, stroke or systemic embolism, bleeding events, and all‐cause death. We accepted the original definitions of the included studies; and (5) follow‐up duration: no restrictions.

Certain publication types (e.g., reviews, case series, case reports, meta‐analyses, editorials, and conference abstracts) or studies with insufficient data were excluded. If the study population had a substantial overlap among different studies, we included the study with the longest follow‐up or largest sample size.

### 2.3 Data extraction

All of the retrieved studies were independently screened by two reviewers. The first phase of screening was performed by reading the titles and abstracts of the records. Then, the second phase of screening involved reviewing the full text of the studies to identify potentially eligible studies. Discrepancies were resolved through discussion or dealt with by consultation with a third reviewer. Ultimately, we included the studies that met the eligibility criteria mentioned above. For each included study, the following basic information was collected: study characteristics (e.g., the first author and publication year, study design, study period), patient characteristics (e.g., age, sex), type of NOACs, type of VKAs, follow‐up time, and outcomes of interest. In the NOAC or VKA groups, the number of events, event rates, and sample size were extracted for the reported outcomes (thrombus resolution, stroke or systemic embolism, bleeding events, and all‐cause death).

### 2.4 Quality assessment

The Newcastle–Ottawa Scale (NOS) tool was used to assess the quality of observational studies. This tool had a total score of nine points. Each included study was awarded a maximum of one point for each numbered item within the selection of cohorts (four points), the comparability of cohorts (two points), and the assessment of the outcome (three points). In this meta‐analysis, we defined an NOS score of ≥6 points and <6 points as moderate‐to‐high quality and low quality, respectively.^6^, ^25^, ^26^

### 2.5 Statistical analysis

The Cochrane Q test and *I* ^2^ statistic were used to assess consistency across the included studies. For the Q statistic, a *p* value of <.1 indicated substantial heterogeneity. For the *I* ^2^ statistic, 25% or less, 50%, and 75% or more indicated low, moderate, and high heterogeneity, respectively. For each study, the number of events and sample size in each treatment group were pooled by a random‐effects model. The pooled treatment effects were expressed as odds ratios (ORs) with 95% confidence intervals (CIs). Sensitivity analysis was performed to examine the influence of each study on the pooled results. We also reperformed the analysis by using a fixed‐effects model. Publication bias was visually assessed by using a funnel plot. All statistical analyses were performed by using Review Manager Version 5.3 (the Nordic Cochrane Center, Rigshospitalet, Denmark; http://ims.cochrane.org/revman). The statistical significance threshold was set at a *p* value of <.05.

## 3 RESULTS

### 3.1 Study selection

**FIGURE 1:**
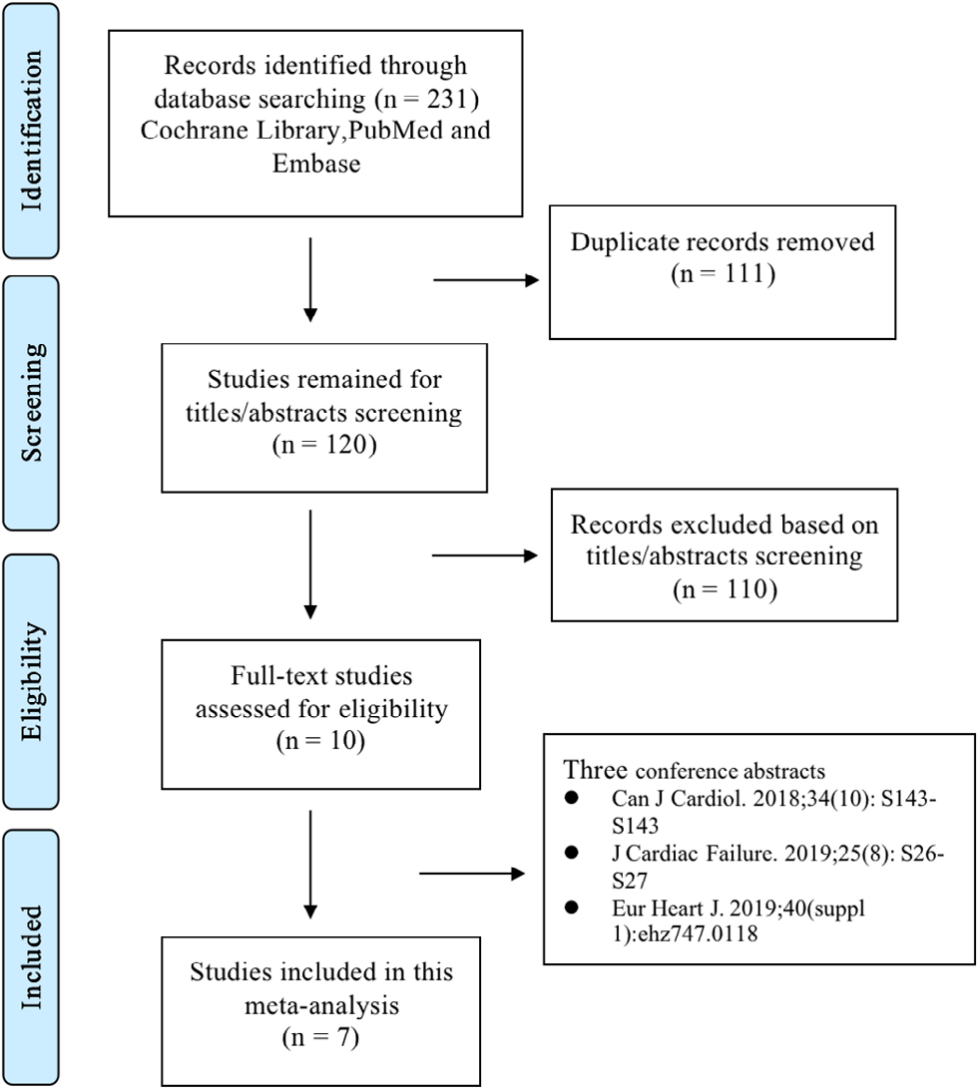
The literature retrieval process of this meta‐analysis

The literature retrieval process of this meta‐analysis is presented in Figure 1. We identified 231 studies through the Cochrane Library, PubMed and Embase electronic databases after we excluded duplicate publications. Based on the title/abstract screenings, 110 studies were excluded according to the predefined criteria. Then, three conference abstracts were excluded based on the full text screenings (Table S1). Finally, a total of seven observational retrospective studies that were published in 2020^18^, ^19^, ^20^, ^21^, ^22^, ^23^, ^24^ involving 865 patients with left ventricular thrombus (*n* = 266 for NOACs and *n* = 599 for VKAs) were included in this meta‐analysis.

### 3.2 Study characteristics

The baseline characteristics of these included studies are shown in Table 1. Five studies used warfarin as the reference, while the combined VKAs, including warfarin, acenocoumarol, and fluindione, were regarded as the control group in the study by Daher et al.^20^ Specifically, Robinson et al.^18^ studied a total of 514 patients diagnosed with left ventricular thrombus on echocardiogram, including 236 patients treated with warfarin only and 121 patients treated with NOACs only. In the study by Jones et al.,^22^ left ventricular thrombus was diagnosed in 101 patients after acute myocardial infarction, which included 60 patients who were started on warfarin and 41 patients who used NOACs. Iqbal et al.^19^ enrolled 84 patients diagnosed with left ventricular thrombus, including 62 patients who received NOACs and 22 who received warfarin. Guddeti et al.^21^ retrospectively identified 99 patients with a diagnosis of left ventricular thrombus (80 patients on warfarin and 19 on NOACs). The retrospective study by Daher et al.^20^ included 59 patients with left ventricular thrombus (42 patients on VKAs and 17 on NOACs). Ali et al.^23^ identified a total of 110 patients with left ventricular thrombus, but only 32 patients with NOACs and 60 patients with warfarin were included in our meta‐analysis. A total of 14 NOAC and 59 VKA patients with left ventricular thrombus were included in the study by Cochran et al.^24^ All seven included studies had an NOS score of ≥6 points (Table S2).

**TABLE 1:** Baseline characteristics of the included studies of this meta‐analysis

Included studies	Type of study	Study period	Sample size (*n*)^a^	Age (year)^b^	Male(%)^b^	Type of NOACs	Patients with reduced NOACs (%)	Type of VKAs	Follow‐up
Robinson et al. (2020)	Retrospective study	October 2013–March 2019	357	58.1/58.2	77.7/72.0	Dabigatran; rivaroxaban; apixaban	NA	Warfarin	Median of 1.0 year
Jones et al. (2020)	Retrospective study	May 2015–December 2018	101	58.7/60.8	80.4/85.0	Rivaroxaban; apixaban; edoxaban	65.7%	Warfarin	Median of 2.2 years
Iqbal et al. (2020)	Retrospective study	December 2012–June 2018	84	62.0/62.0	91.0/89.0	Dabigatran; rivaroxaban; apixaban	40.9%	Warfarin	Mean of 3.0 years
Guddeti et al. (2020)	Retrospective study	January 2012–March 2019	99	60.7/61.3	79.0/68.8	Dabigatran; rivaroxaban; apixaban	NA	Warfarin	1.0 year
Daher et al. (2020)	Retrospective study	January 2010–August 2019	59	57.0/61.0	82.4/83.0	Dabigatran; rivaroxaban; apixaban	NA	Warfarin; acenocoumarol; fluindione	NA
Ali et al. (2020)	Retrospective study	NA	92	59.2/58.0	81.3/98.3	Dabigatran; rivaroxaban; apixaban; edoxaban	NA	Warfarin	1.0 year
Cochran et al. (2020)	Retrospective study	January 2014–December 2017	73	51.5/62.0	78.6/76.3	Dabigatran; rivaroxaban; apixaban; edoxaban	NA	Warfarin	1.0 year

Abbreviations: NOAC, non‐vitamin K antagonist oral anticoagulant; VKAs, vitamin K antagonists; NA, not available.

^a^ Patients treated with NOACs plus those with VKAs.

^b^ NOACs/VKAs.

### 3.3 Effect of NOACs versus that of VKAs on thrombus resolution

**FIGURE 2:**
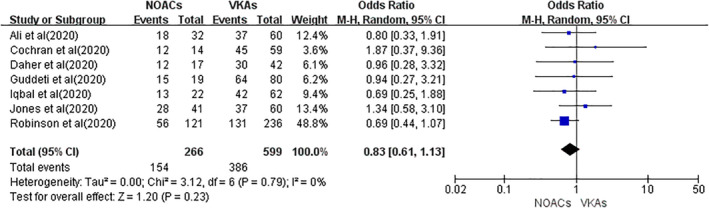
The outcome of thrombus resolution between NOACs versus VKAs. Abbreviations: OR, odds ratio; CI, confidence interval; NOACs, non‐vitamin K antagonist oral anticoagulants; VKAs, vitamin K antagonists

All seven included studies reported the rate of thrombus resolution. A total of 154 events were found in 266 patients with NOACs, and 386 events were observed in 599 patients with VKAs. As shown in Figure 2, a random‐effects model analysis suggested no difference in the rate of thrombus resolution between the NOAC and VKA groups (OR = 0.83, 95% CI 0.61–1.13; *p* = .23). No heterogeneity was found across the included studies (Q statistic: *p* = .79, and *I* ^2^ = 0%).

### 3.4 Effect of NOACs versus that of VKAs on stroke or systemic embolism

**FIGURE 3:**
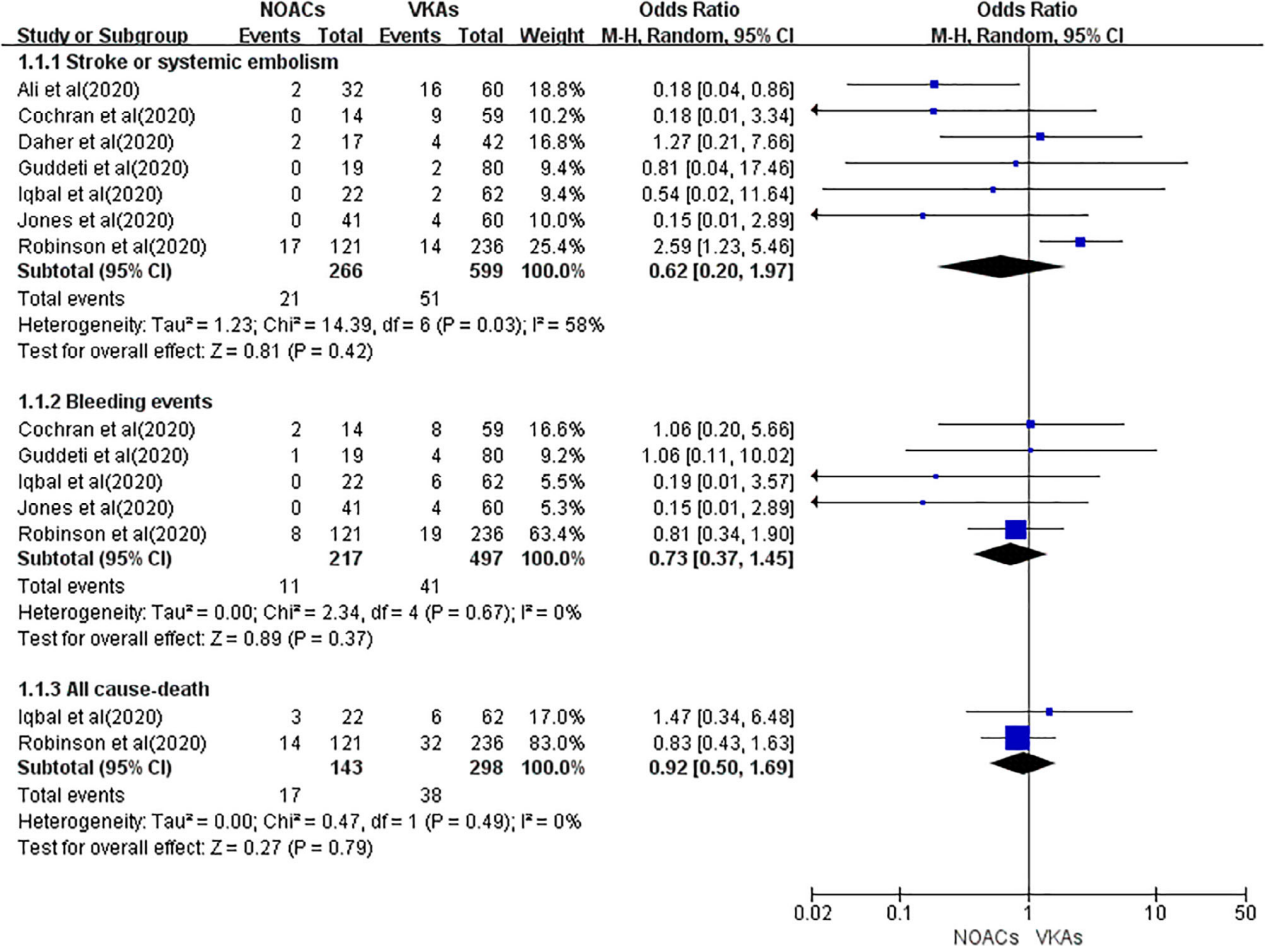
The outcomes of stroke or systemic embolism, bleeding events, and all‐cause death between NOACs versus VKAs. Abbreviations: OR, odds ratio; CI, Confidence interval; NOACs, non‐vitamin K antagonist oral anticoagulants; VKAs, vitamin K antagonists

All seven included studies reported the event rate of stroke or systemic embolism. As shown in Figure 3, a total of 21 and 42 events were found in 266 NOAC users and 599 VKA users, respectively. In the pooled analysis, we found no significant difference in the event rate of stroke or systemic embolism between the two studied groups treated with NOACs versus VKAs (OR = 0.62, 95% CI 0.20–1.97; *p* = .42), with moderate heterogeneity (Q statistic: *p* = .03 and *I* ^2^ = 58%).

### 3.5 Effect of NOACs versus that of VKAs on bleeding events

A total of five included studies assessed the effect of NOACs versus that of VKAs on bleeding risks. In the study by Guddeti et al., bleeding events, defined as any life‐threatening bleeding, drop in hemoglobin ≥2 g, and/or bleeding requiring hospitalization or evaluation by endoscopy, were observed in five patients. Iqbal et al. used clinically relevant bleeding and observed three episodes of gastrointestinal bleeding (one requiring hospitalization or blood transfusion) and three epistaxis events (0 requiring hospitalization or blood transfusion). Jones et al. defined bleeding events using the BARC criteria, and they only presented the specific data of major bleeding events (BARC >2). Robinson et al. reported bleeding events requiring cessation in anticoagulation. Cochran et al. included minimal, minor, and major bleeding assessed by Thrombolysis in Myocardial Infarction bleeding criteria. As shown in Figure 3, the pooled data suggested no difference in the rate of bleeding events between NOACs and VKAs (OR = 0.73, 95% CI 0.37–1.45; *p* = .37). We found no heterogeneity (Q statistic: *p* = .67, and *I* ^2^ = 0%).

### 3.6 Effect of NOACs versus that of VKAs on all‐cause death

Two studies assessed the effect of NOACs versus that of VKAs on all‐cause death. A random‐effects model analysis suggested that the event rate of all‐cause death between NOACs and VKAs was not significantly different (OR = 0.92, 95% CI 0.50–1.69; *p* = .79), with no heterogeneity of this part (Q statistic: *p* = .49, and *I* ^2^ = 0%). The results of this part should be interpreted cautiously due to the limited number of included studies.

### 3.7 Sensitivity analysis

After exclusion of one study at a time, the corresponding results were not changed substantially. We only included the six included studies that used warfarin as the reference and reperformed the aforementioned analyses. Pooling data from these studies showed no differences between NOACs and warfarin in the event rates of thrombus resolution, stroke or systemic embolism, bleeding events, or all‐cause death (all *p* > .05; Figures S1 and S2). In addition, we also reperformed the analysis with a fixed‐effects model, which suggested that NOACs versus VKAs yielded nonsignificantly different risks for the outcomes of thrombus resolution, stroke or systemic embolism, bleeding events, and all‐cause death (Figures S3 and S4).

### 3.8 Publication bias

The publication bias assessed by using funnel plots is shown in Figures S5 and S6. Of note, according to the Cochrane handbook, it is theoretically unsuitable to assess publication bias for the reported effect estimates when the number of included studies is less than 10. Therefore, the results of the publication bias shown by the funnel plots should be interpreted cautiously.

## 4 DISCUSSION

n the present study, the pooling results from the seven included studies showed that in patients with left ventricular thrombus, there were similar rates of effectiveness and safety outcomes, including stroke or systemic embolism, bleeding events, and all‐cause death, between treatment with NOACs and VKAs. In addition, we did not observe significant differences in thrombus resolution between use of NOACs versus VKAs for oral anticoagulation. Specifically, there were no differences in the event rates of thrombus resolution, stroke or systemic embolism, bleeding events, or all‐cause death between NOAC and warfarin treatments. Subsequent randomized controlled trials are warranted to further demonstrate the effectiveness and safety of NOACs and VKAs for this indication.

Left ventricular thrombus is a major complication of several cardiac diseases, such as heart failure, acute myocardial infarction, and cardiomyopathy, resulting in high risk of major adverse cardiovascular events and mortality. The potential mechanisms of thrombus genesis may be due to reduced cardiac output, increased left ventricular volumes, insufficient contractility, and regional wall motion abnormalities. For myocardial infarction‐related ventricular thrombus, endocardial surface abnormalities after myocardial infarction (e.g., fibrosis and inflammatory or infiltrative alterations) could result in blood stasis. Moreover, for heart failure‐related ventricular thrombus, the hypercoagulable state could accelerate the formation of thrombi. Anticoagulation therapy is required to prevent the risk of stroke and its subsequent mortality in the population of patients with left ventricular thrombi. Indeed, antithrombotic therapy has been found to reduce the risk of mortality in patients with left ventricular thrombus.^4^

Previously, VKAs have often been used to treat left ventricular thrombi. NOACs have been widely used in patients with venous thromboembolism and AF.^27^ However, data comparing the off‐label use of NOACs for the treatment of left ventricular thrombus are still limited. In our current study, we performed a systematic review and meta‐analysis of seven observational studies comparing the effectiveness and safety of NOACs and VKAs on thrombus resolution and clinical outcomes in patients with left ventricular thrombus. We included 993 patients, of whom nearly 70% received VKAs (predominantly warfarin) and 30% received a NOAC. Our results after pooling the existing data suggested that NOACs had effectiveness (as assessed by thrombus resolution, stroke or systemic embolism, bleeding events, and all‐cause death) and safety (as assessed by bleeding events) similar to those of VKAs in treating left ventricular thrombus. Our findings were consistent with a prior meta‐analysis by Cochran et al. However, in the study by Cochran et al.,^24^ three of their included studies were published conference abstracts, limiting the validity of the corresponding findings. Based on recent evidence, NOACs at least potentially provide a more convenient alternative for the management of left ventricular thrombus than warfarin because of their ease of administration, absence of monitoring of anticoagulant activity, and fewer drug–drug or drug–food interactions.

In our meta‐analysis, NOACs had safety similar to that of VKAs in patients with left ventricular thrombus. However, our results should be interpreted with caution in the context of available evidence, and the small sample size cannot be ignored. A randomized controlled trial is warranted to further assess the safety of NOACs in patients with left ventricular thrombus. Four ongoing randomized clinical trials (NCT 02982590, NCT03232398, NCT03926780, and NCT03764241) may provide more comprehensive insights for this purpose.

Considering the observational nature of the included studies, our data from this meta‐analysis were still limited. Until the results from randomized control trials designed to examine the effect of NOACs versus VKAs are available with respect to patients with left ventricular thrombus, our evidence could not provide reliable guidance on the choice of NOACs versus VKAs in real‐life patients. Further prospective trials are needed to clarify whether there are more benefits from NOACs than from VKAs for this population. Moreover, the findings in the present meta‐analysis were driven by combining different types or doses of NOACs. Due to the limited data, we did not perform a subgroup analysis based on the type or dose of NOAC. Because of the widespread use of NOACs in clinical practice, further study should take the type or dose of NOAC into consideration.

## 5 LIMITATIONS

Several limitations should be acknowledged in this meta‐analysis. First, with respect to the observational retrospective nature of this meta‐analysis, residual confounders might exist. Further study could include propensity score‐matched or multivariate adjusted effect estimates to confirm our findings. Second, the rates of some outcomes in the pooled analysis had quite wide CIs, which might be largely due to the limited sample size (five of seven included studies involved fewer than 100 patients) and small number of events. Further large‐scale studies with a better design are needed to confirm our results. Third, since small numbers of patients treated with NOACs were included, it was impossible to evaluate the effectiveness and safety of individual NOACs versus VKAs in patients with left ventricular thrombus. Fourth, among VKA users, the time in the therapeutic range (TTR) was not considered because only one included study by Jones et al. compared NOACs versus VKAs with a TTR ≥65%. Finally, the underlying etiology of left ventricular thrombus discovery was different across the included studies.

## 6 CONCLUSIONS

Based on previously published studies, our current evidence suggests that NOACs and VKAs have similar rates of thrombus resolution and clinical outcomes among patients with left ventricular thrombus. Further large‐scale studies with a better design should confirm our results. Clinicians should continue to examine the off‐label use of NOACs in the treatment of left ventricular thrombus.

## CONFLICT OF INTEREST

The authors declare that they have no conflict of interest.

## AUTHOR CONTRIBUTIONS

Shunhui Li and Xiaojie Jiang: Data curation; Shunhui Li and Jing Hu: Formal analysis; **Shunhui Li and Qiangzhen Xiong**: Investigation; **Shunhui Li and Yuqing Deng**: Methodology; **Shunhui Li and Yuqing Deng**: Software; **Liyun Liu and Hui Chen**: Supervision; **Shunhui Li, Tao He, and Xiaojie Jiang**: Validation; **Shunhui Li**: Writing – original draft; **Liyun Liu and Hui Chen**: Writing – review & editing.

## Supporting information


**Data S1.** Supporting Information.
