# Supplementary material for: Assessment of non‐vitamin K antagonist oral anticoagulants for the management of left ventricular thrombus
Source: Clin Cardiol. 2021 Apr 2;44(6):754–60. doi: 10.1002/clc.23553 (PMC8207971; doi:10.1002/clc.23553)
Supplement: Supplementary file 1 — Data S1. Supporting Information. [file CLC-44-754-s001.docx]

**Supplemental Table 1. A total of 3 published abstracts excluded in this meta-analysis**

| **NO.** | **Excluded studies** |
| --- | --- |
| 1 | Jaidka A, Zhu T, Lavi S, Johri A. Treatment of left ventricular thrombus using warfarin versus direct oral anticoagulants following anterior myocardial infarction. Can J Cardiol. 2018;34(10): S143-S143. |
| 2 | Bass M, Page RL, Kiser TH, et al. Comparative effectiveness of direct oral anticoagulants and warfarin for the treatment of left ventricular thrombus. J Cardiac Failure. 2019;25(8): S26-S27. |
| 3 | Gama F, Freitas P, Trabulo M, et al. 459Direct oral anticoagulants are an effective therapy for left ventricular thrombus formation.Eur Heart J. 2019;40(suppl 1):ehz747.0118. |

**Supplemental Table 2. Quality assessment of the included studies of this meta-analysis**

| **Included studies** | **Selection** | | | | **Comparability** | **Outcome** | | | **Total** |
| --- | --- | --- | --- | --- | --- | --- | --- | --- | --- |
|  | **Exposed**  **cohort** | **Non-exposed**  **cohort** | **Ascertainment**  **of exposure** | **Outcome**  **of interest** |  | **Assessment of**  **outcome** | **Length of**  **follow-up** | **Adequacy of**  **follow up** |  |
| Robinson et al(2020) | **＊** | **＊** | **＊** | **＊** | **＊＊** | **＊** | **＊** | **＊** | 9 |
| Jones et al(2020) | **＊** | **＊** | **＊** | **＊** | **＊＊** | **＊** | **＊** | **＊** | 9 |
| Iqbal et al(2020) | **＊** | **＊** | **＊** | **＊** | **＊＊** | **＊** | **＊** | **＊** | 9 |
| Guddeti et al(2020) | **＊** | **＊** | **＊** | **＊** | **＊** | **＊** | **＊** | **＊** | 8 |
| Daher et al(2020) | **＊** | **＊** | **＊** | **＊** | **＊** | **＊** | **＊** | **-** | 7 |
| Ali et al(2020) | **＊** | **＊** | **＊** | **＊** | **＊** | **＊** | **＊** | **-** | 7 |
| Cochran et al(2020) | **＊** | **＊** | **＊** | **＊** | **＊** | **＊** | **＊** | **-** | 7 |


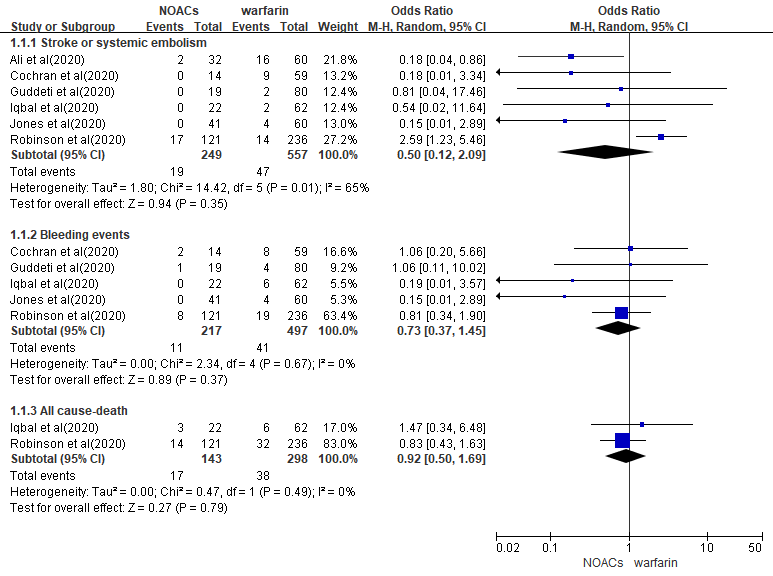


**Supplemental Figure 1. The outcomes of stroke or systemic embolism, bleeding events, and all-cause death between NOACs versus warfarin**


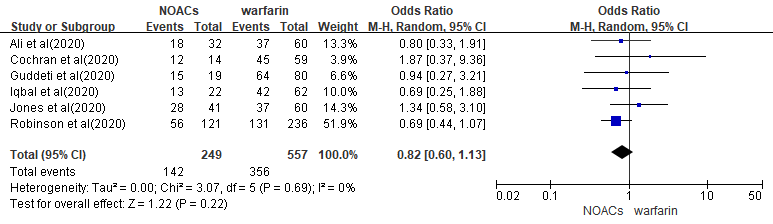


**Supplemental Figure 2. The outcome of thrombus resolution between NOACs versus warfarin**


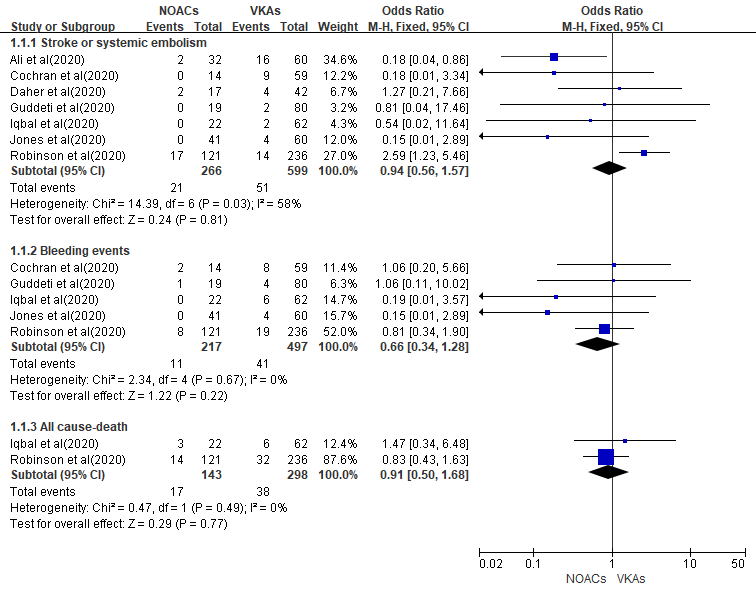


**Supplemental Figure 3. The outcomes of stroke or systemic embolism, bleeding events, and all-cause death between NOACs versus VKAs (fixed-effects model)**


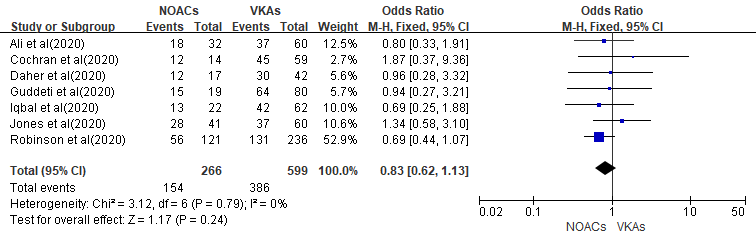


**Supplemental Figure 4. The outcome of thrombus resolution between NOACs versus VKAs (fixed-effects model)**


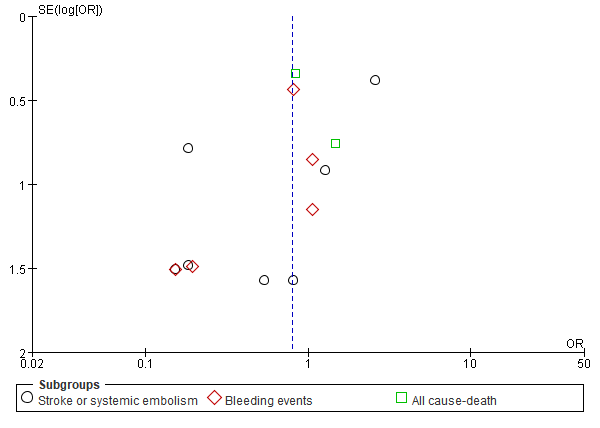


**Supplemental Figure 5. The funnel plot of stroke or systemic embolism, bleeding events, and all-cause death between NOACs versus VKAs**


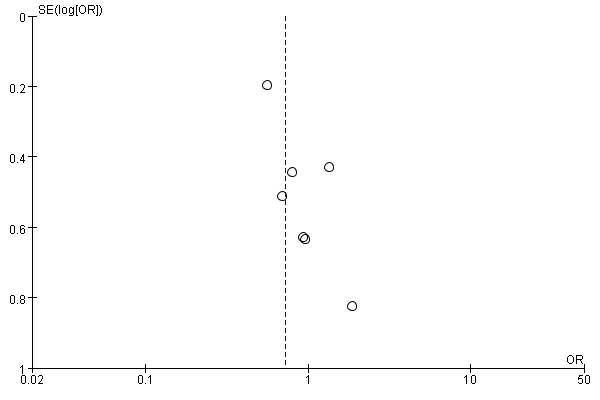


**Supplemental Figure 6. The funnel plot of thrombus resolution between NOACs versus VKAs**
